# Chemical Stability of High-Entropy Spinel in a High-Pressure Pure Hydrogen Atmosphere

**DOI:** 10.3390/ma17133309

**Published:** 2024-07-04

**Authors:** Kamil Domaradzki, Anna Adamczyk, Michał Pyzalski, Tomasz Brylewski, Marek Nowak, Mieczysław Jurczyk

**Affiliations:** 1 Faculty of Materials Science and Ceramics, AGH University of Krakow, al. Mickiewicza 30, 30-059 Krakow, Poland; domar@agh.edu.pl (K.D.); aadamcz@agh.edu.pl (A.A.); michal.pyzalski@agh.edu.pl (M.P.); brylew@agh.edu.pl (T.B.); 2 Institute of Materials Science and Engineering, Faculty of Materials Engineering and Technical Physics, Poznan University of Technology, Jana Pawla II 24, 61-138 Poznań, Poland; marek.nowak@put.poznan.pl; 3 Institute of Material and Biomedical Engineering, Faculty of Mechanical Engineering, University of Zielona Góra, 65-417 Zielona Góra, Poland

**Keywords:** high-entropy oxide, spinel structure, mechanochemical synthesis, hydrogen

## Abstract

This paper focuses on high-entropy spinels, which represent a rapidly growing group of materials with physicochemical properties that make them suitable for hydrogen energy applications. The influence of high-pressure pure hydrogen on the chemical stability of three high-entropy oxide (HEO) sinter samples with a spinel structure was investigated. Multicomponent HEO samples were obtained via mechanochemical synthesis (MS) combined with high-temperature thermal treatment. Performing the free sintering procedure on powders after MS at 1000 °C for 3 h in air enabled achieving single-phase (Cr_0.2_Fe_0.2_Mg_0.2_Mn_0.2_Ni_0.2_)_3_O_4_ and (Cu_0.2_Fe_0.2_Mg_0.2_Ni_0.2_Ti_0.2_)_3_O_4_ powders with a spinel structure, and in the case of (Cu_0.2_Fe_0.2_Mg_0.2_Ti_0.2_Zn_0.2_)_3_O_4_, a spinel phase in the amount of 95 wt.% was achieved. A decrease in spinel phase crystallite size and an increase in lattice strains were established in the synthesized spinel powders. The hydrogenation of the synthesized samples in a high-pressure hydrogen atmosphere was investigated using Sievert’s technique. The results of XRD, SEM, and EDS investigations clearly showed that pure hydrogen at temperatures of up to 250 °C and a pressure of up to 40 bar did not significantly impact the structure and microstructure of the (Cr_0.2_Fe_0.2_Mg_0.2_Mn_0.2_Ni_0.2_)_3_O_4_ ceramic, which demonstrates its potential for application in hydrogen technologies.

## 1. Introduction

Entropy-stabilized materials have been intensively investigated since 2004, when Cantor and Yeh presented, for the first time, multi-principle element alloys built of a simple single-phase structure that formed due to high configurational entropy and thus were named high-entropy alloys (HEAs) [1,2]. The discovery of HEAs led to the development of the concept of creating other multi-principle component systems, the phase composition of which is stabilized by high configurational entropy. Consequently, a new group of materials, designated as high-entropy ceramics, was developed [3], which includes high-entropy borides (HEBs) [4], high-entropy carbides (HECs) [5,6], high-entropy nitrides (HENs) [5], and most notably, high-entropy oxides (HEOs) [7]. Structure stabilization is characteristic of all the aforementioned ceramic materials. In other words, each of the high-entropy ceramics consists of a solid solution phase because of the high configurational entropy in each system the structure constitutes. To achieve these thermodynamic properties, it is necessary to mix several components (usually at least five) in an equimolar or near equimolar ratio. The large number of metal ions allows for the aforementioned cations to assume random positions throughout the entire crystalline structure of a given high-entropy material. This results in novel properties, due to the synergetic interactions between different ion pairs.

In 2015, Rost et al. obtained the first high-entropy oxide (HEOx) with an equimolar composition (Co,Cu,Mg,Ni,Zn)O via the solid-state reaction method. This oxide was a single-phase solid solution, with a rock salt structure of (Fm-3m) cubic symmetry [7]. This was the beginning of dynamic development for this group of materials. Up to now, aside from rock salt-structured HEOx, many different high-entropy structures have been identified, such as materials with the following structures: spinel [8,9], perovskite [10,11,12,13], pyrochlore [14,15,16,17], Aurivillius [18], monoclinic [19], bixbyite [20], and granite [21].

Among a wide group of high-entropy oxide materials, spinels deserve special attention due to several unique physicochemical properties that have not been previously found in conventional spinels. In 2018, Dąbrowa et al. synthesized, for the first time, a spinel-type HEOx with the composition (CoCrFeMnNi)_3_O_4_ and, subsequently, single-phase (CrFeMgMnNi)_3_O_4_ and (CoCrFeMgMn)_3_O_4_ spinels [8,9]. Spinel-structured oxides are a class of materials with the general composition of AB_2_O_4_, which demonstrates oxide ion placement in a cubic, densely packed structure, with divalent cations in tetrahedral positions and trivalent cations in octahedral positions. HEOx spinels have a high potential for application, as they pertain to manufacturing materials for energy conversion and storage, due to their unique electron structure, which is the result of the diverse compositions and valence states of their metal cations. HEOx spinel materials are much better candidates for anode materials in Li ion batteries than HEOx with rock salt structures because of their significantly higher Li reversible capacity [22,23]. HEOx spinels are primarily used for lithium-ion storage, and the current focus is on improving oxygen vacancies [24]. Increasing oxygen vacancies can enhance the electrical conductivity for O atoms in general, as well as compensate for the initial energy band structure while the conduction band moves to the Fermi level [25,26].

Experimental results from literature up to now have demonstrated the possibility of producing single-phase entropy-stabilized oxides using methods other than solid-state reaction [26,27,28,29,30,31]. Sarkar et al. [29] obtained (Co,Mg,Ni,Zn)O and (Co,Cu,Mg,Ni,Zn)O oxides with the rock salt structure via flame spray pyrolysis (FSP) and reverse co-precipitation (RCP). Sarkar’s team was also able to directly synthesize a HEOx without the necessity for further thermal treatment using the nebulized spray pyrolysis (NSP) technique. Conversely, in the case of the FSP and RCP methods, further thermal treatment of the samples was required to receive their single-phase structure. Other studies showed that synthesis in a single stage without having to perform further technological procedures, resulting in a single-phase (Co,Cu,Mg,Ni,Zn)O oxide, is possible using solution combustion synthesis (SCS) [21]. To synthesize polycrystalline HEOx with nanometric grain sizes, the above-mentioned powder preparation techniques can be successfully applied, along with other wet chemistry methods, including co-precipitation and hydrothermal synthesis [27], and the citrate gel method [30]. In these methods, synthesis occurs in a solution and also requires energy input to overcome the activation barrier. Such an approach often leads to losses associated with the ways heat spreads out. One of the most efficient methods of distributing energy to a reaction system is mechanosynthesis (MS) [32,33,34]. As it turns out, energy provided to a system consisting of a few powdered solids in the form of mechanical force can be sufficient to form new bonds or break the existing ones. Thus, mechanochemistry can be applied in the synthesis of simple non-organic compounds, crystalline structure reorganization (transitions between polymorphic phases), layer structure re-organization, and, also, organic compound synthesis. A single-phase HEOx has been obtained via this technique at room temperature [35].

An energy carrier in the form of hydrogen has become the best currently considered alternative to fossil fuels because it is three times more energetically efficient than conventional fuels. The application of hydrogen as a fuel opens the door to several possibilities when it comes to satisfying the world’s energy needs. Moreover, it can constitute a way to store excess energy originating from renewable energy sources, such as solar or wind energy. The most ecological method for generating hydrogen is water electrolysis facilitated by electrical energy from renewable sources. Apparatuses that use this process are called electrolyzers, and hydrogen obtained in this manner has been designated as “green hydrogen” because carbon dioxide emission does not occur during its production. However, hydrogen cannot become widely adopted as a fuel without first developing state-of-the-art materials for hydrogen technologies, including materials capable of stable operation in gaseous hydrogen environments. High-entropy spinels can be included among materials considered for this purpose, due to their interesting physicochemical properties.

So far, chemical stability studies have only been carried out in a high-pressure hydrogen atmosphere for nanocrystalline HEOx with an equimolar composition of (Co_0.2_Cu_0.2_Mg_0.2_Ni_0.2_Zn_0.2_)O, which was directly synthesized using mechanochemical synthesis performed in a protective argon atmosphere, and also for sinter obtained from the aforementioned nanopowder after thermal treatment at 1000 °C for 5 h in air [36]. Investigations demonstrated that the high-entropy rock salt structure of the studied nanopowder is not fully stable when exposed to hydrogen at 250 °C and a pressure of up to 40 bar. Conversely, when the (Co_0.2_Cu_0.2_Mg_0.2_Ni_0.2_Zn_0.2_)O sinter was exposed to similar conditions, the reducing atmosphere did not significantly impact the structure or microstructure of the material [36].

For a HEOx to be applied in hydrogen technologies, its physicochemical properties must remain stable in hydrogen-containing environments. Thus, further research is required to investigate the effect of hydrogen on the aforementioned spinel oxide properties.

The goal of this work was to synthesize three high-entropy spinels with the following compositions: (Cr_0.2_Fe_0.2_Mg_0.2_Mn_0.2_Ni_0.2_)_3_O_4_, (Cu_0.2_Fe_0.2_Mg_0.2_Ti_0.2_Zn_0.2_)_3_O_4_ and (Cu_0.2_Fe_0.2_Mg_0.2_Ni_0.2_Ti_0.2_)_3_O_4_ using mechanochemical synthesis (MS) combined with high-temperature thermal treatment and, subsequently, to study the influence of high-pressure pure hydrogen on the chemical stability of the three obtained HEOx sinter samples. Hydrogenation of the synthesized samples was performed at 250 °C and with a pressure of up to 40 bar using Sievert’s technique. On the other hand, the phase and chemical compositions, along with the morphology of the obtained spinel samples, were identified via XRD and SEM-EDS techniques.

## 2. Materials and Methods

Three equimolar high-entropy oxides (HEOs), i.e., (Cr_0.2_Fe_0.2_Mg_0.2_Mn_0.2_Ni_0.2_)_3_O_4_, (Cu_0.2_Fe_0.2_Mg_0.2_Ti_0.2_Zn_0.2_)_3_O_4_, and (Cu_0.2_Fe_0.2_Mg_0.2_Ni_0.2_Ti_0.2_)_3_O_4_, were obtained using mechanochemical synthesis (MS) combined with thermal treatment. The following initial powders were used for synthesis: magnesium oxide (Chempur, Piekary Śląskie, Poland, p.a.), zinc oxide (Chempur, p.a.), nickel oxide (Acros Organics, Poznań, Poland, 97%), copper (II) oxide (Acros Organics, >99%), iron (III) oxide (Chempur, p.a.), titanium (IV) oxide (Merck, Poznań, Poland, 99.9%), manganese oxide (MnO_x_) (Chempur, p.a.), and chromium (III) oxide (P.O.Ch., Gliwice, Poland, p.a.). Mixtures of the respective powders for a given composition were prepared using a high-energy SPEX 8000 Mixer/Mill (SPEX SamplePrep, Metuchen, NJ, USA) in a steel chamber filled with steel grinders. The ball-to-powder ratio (BPR) was 5:1. The milling process was performed in an air atmosphere for 24 h in the cases of (Cr_0.2_Fe_0.2_Mg_0.2_Mn_0.2_Ni_0.2_)_3_O_4_ and (Cu_0.2_Fe_0.2_Mg_0.2_Ti_0.2_Zn_0.2_)_3_O_4_ and for 72 h in the case of (Cu_0.2_Fe_0.2_Mg_0.2_Ni_0.2_Ti_0.2_)_3_O_4_. The three powders received after the mechanochemical synthesis process were subjected to thermal treatment at 1000 °C for 3 h in air, after which the resulting sinters were rapidly cooled down to room temperature.

The crystal structure of the samples was investigated via XRD, using a PANalytical Empyrean diffractometer with CuKα (λ = 1.54056 Å) radiation. HighScore 5.2 version with Plus option software was used to conduct a quantitative analysis that included identifying lattice constants and mass fractions of the detected phases via Rietveld refinement, as well as identifying crystallite size with the help of the Williamson–Hall method, using the Cauchy function to determine both crystallite size and lattice distortions. The morphology of the sintered powders was studied via SEM-EDS using an Apreo 2S high-resolution scanning electron microscope (ThermoFisher Scientific, Waltham, MA, USA). Observations were made using an in-column detector, under high vacuum conditions, at an accelerating voltage of 5–15 KV. Qualitative and quantitative analyses were performed by a standardless method using an SDD detector from an EDAX Octane Elite system with APEX™ Advanced 2.5 software. The obtained spinels were hydrogenated under high pressure using a conventional, fully automated Sievert’s apparatus (Particulate Systems, HPVA 200, Norcross, GA, USA) operating at 250 °C.

## 3. Results

### 3.1. Mechanochemical Synthesis of Spinel HEOx

**Figure 1:**
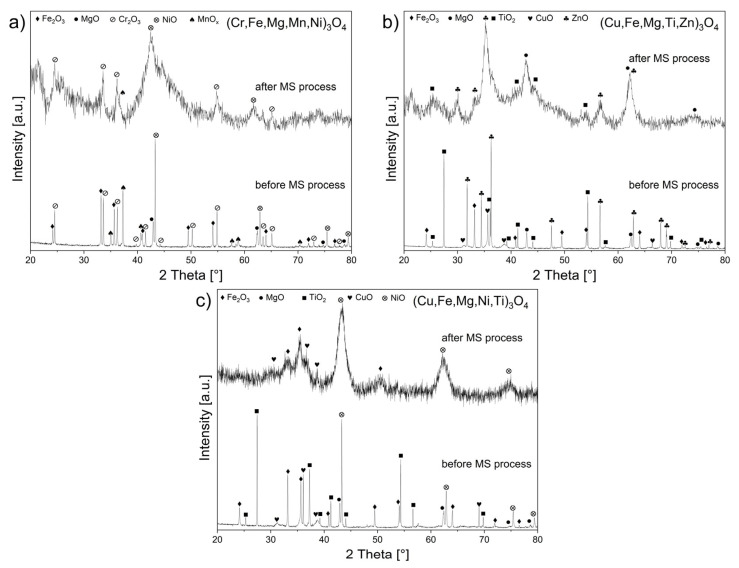
XRD diffractograms of samples: (**a**) (Cr,Fe,Mg,Mn,Ni)_3_O_4_, (**b**) (Cu,Fe,Mg,Ti,Zn)_3_O_4_ (after 24 h of the MS process), and (**c**) (Cu,Fe,Mg,Ni,Ti)_3_O_4_ (after 72 h of the MS process).

Figure 1 presents X-ray diffractograms for powders with the following nominal compositions: (Cr,Fe,Mg,Mn,Ni)_3_O_4_ (Figure 1a), (Cu,Fe,Mg,Ti,Zn)_3_O_4_ (Figure 1b), and (Cu,Fe,Mg,Ni,Ti)_3_O_4_ (Figure 1c), which were obtained after the mechanochemical synthesis process. Moreover, for comparison purposes, X-ray diffractograms of the initial powders were included in these figures.

These results showed that the presence of an (Fd-3m) space group spinel phase was not identified in any of the powders obtained after the MS process, and reflexes originating from phases attributed to NiO, ZnO, Fe_2_O_3_, CuO, Cr_2_O_3_, MnO_x_, MgO, and TiO_2_ were determined.

### 3.2. Spinel HEOx after Hydrogenation

**Figure 2:**
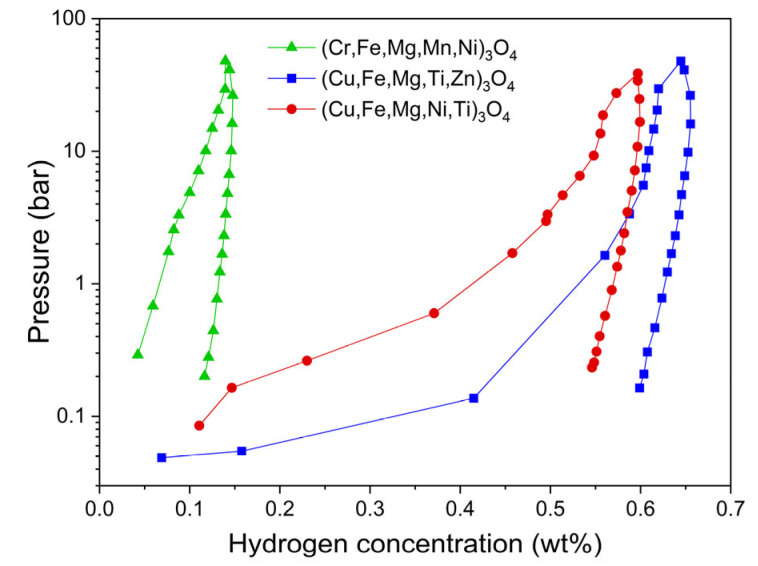
PCT curves plotted for (Cr,Fe,Mg,Mn,Ni)_3_O_4_, (Cu,Fe,Mg,Ti,Zn)_3_O_4_, and (Cu,Fe,Mg,Ni,Ti)_3_O_4_ spinel HEOs during hydrogenation at 250 °C.

Figure 2 illustrates PCT (pressure–composition–temperature) curves for (Cr,Fe,Mg,Mn,Ni)_3_O_4_, (Cu,Fe,Mg,Ti,Zn)_3_O_4,_ and (Cu,Fe,Mg,Ni,Ti)_3_O_4_ spinels obtained after thermal treatment at 1000 °C for 3 h in air. The high-pressure hydrogenation process was performed at 250 °C and with a pressure of up to 40 bar. From these data, it followed that (Cr,Fe,Mg,Mn,Ni)_3_O_4_ demonstrated resistance against the hydrogenation process because hydrogen concentration in that sample was barely 0.14 wt.%. As for the two other spinels, higher hydrogen contents were determined, i.e., 0.64 and 0.60 wt.% for (Cu,Fe,Mg,Ti,Zn)_3_O_4_ and (Cu,Fe,Mg,Ni,Ti)_3_O_4_, respectively.

**Figure 3:**
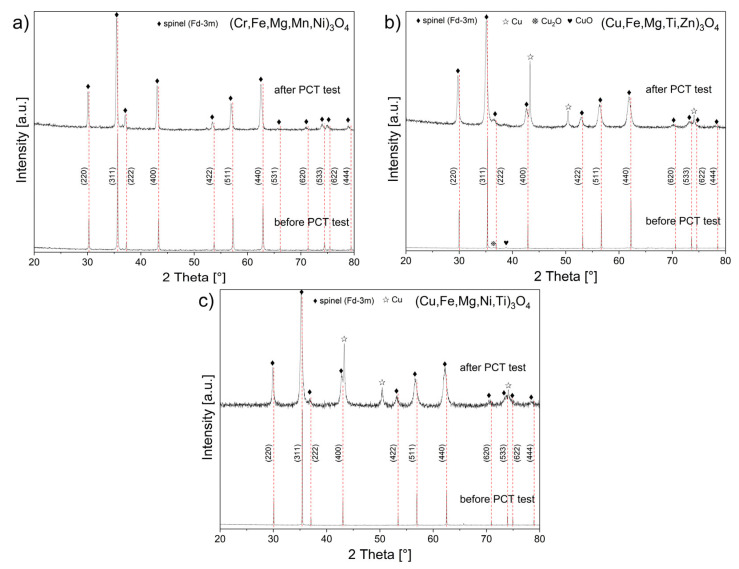
XRD patterns recorded for (**a**) (Cr,Fe,Mg,Mn,Ni)_3_O_4_, (**b**) (Cu,Fe,Mg,Ti,Zn)_3_O_4_, and (**c**) (Cu,Fe,Mg,Ni,Ti)_3_O_4_ spinels samples before and after the PCT test at 250 °C.

The XRD patterns shown in Figure 3 were obtained from HEOx (Cr,Fe,Mg,Mn,Ni)_3_O_4_, (Cu,Fe,Mg,Ti,Zn)_3_O_4_, and (Cu,Fe,Mg,Ni,Ti)_3_O_4_ spinel powders, which were received after high-pressure hydrogenation. These powders were previously sintered at 1000 °C for 3 h in air. For comparison purposes, diffractograms of the aforementioned powders after sintering but before high-pressure hydrogenation were also illustrated.

From the XRD patterns (Figure 3) and data listed in Table 1, it follows that sintering spinel powders, obtained from mechanochemical synthesis (MS) at 1000 °C for 3 h, resulted in 100% spinel phase HEOx for the (Cr,Fe,Mg,Mn,Ni)_3_O_4_ and (Cu,Fe,Mg,Ni,Ti)_3_O_4_ samples and more than 95% of that phase in the case of (Cu,Fe,Mg,Ti,Zn)_3_O_4_. Secondary structures were identified in the (Cu,Fe,Mg,Ti,Zn)_3_O_4_ sample in the form of CuO and Cu_2_O, the summary mass contribution of which was equal to 4.2 wt.%. The unit cell “*a*” parameters in Table 1 for a regular spinel phase are in agreement with the literature data [8,9].

**Figure 4:**
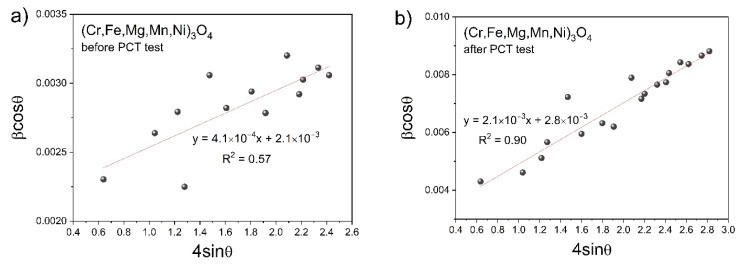
Williamson–Hall plot for (Cr,Fe,Mg,Mn,Ni)_3_O_4_: (**a**) before and (**b**) after hydrogenation at 250 °C.

Data in Table 1 indicate that high-pressure hydrogenation led to an increase in the lattice parameter “*a*” of a regular spinel unit cell, regardless of the presence or lack of additional phases (containing Cu) in each of the studied samples. Furthermore, as a result of powder hydrogenation, the spinel phase crystallite size decreased, and the lattice strains increased. The most minor change in these two parameters was observed in the case of (Cr,Fe,Mg,Mn,Ni)_3_O_4_, which did not undergo reduction under the influence of hydrogen. Both spinels containing copper in their structure, i.e., (Cu,Fe,Mg,Ti,Zn)_3_O_4_ and (Cu,Fe,Mg,Ni,Ti)_3_O_4_, were chemically unstable during high-pressure hydrogenation, and consequently, partial reduction occurred, resulting in the release of metallic copper. As an example, Williamson–Hall plots for the (Cr,Fe,Mg,Mn,Ni)_3_O_4_ sample are presented in Figure 4 before (Figure 4a) and after hydrogenation at 250 °C (Figure 4b).

It can be seen that the coefficient of determination R^2^ for a well-crystallized sample was much lower than in the case of the respective sample after the hydrogenation process, which contained significantly smaller crystallites.

**Figure 5:**
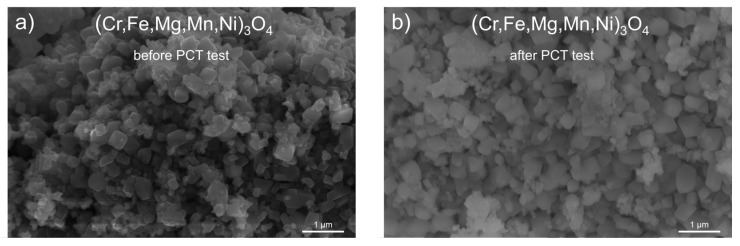
SEM microphotographs for the (Cr,Fe,Mg,Mn,Ni)_3_O_4_ sample obtained: (**a**) before the PCT and (**b**) after the PCT test.

In subsequent studies, an attempt was made to characterize the morphological build of HEOx spinel powders after the high-pressure hydrogenation procedure. For comparison, the morphology of powders before hydrogenation was also observed. Figure 5 illustrates SEM microphotographs of the (Cr,Fe,Mg,Mn,Ni)_3_O_4_ HEOx spinel powder before the PCT test (Figure 5a) and after the PCT test, performed at 250 °C (Figure 5b). The spinel grains in the discussed powder exhibited a regular shape. The sizes of the grains before and after hydrogenation did not notably change and were in the range of ~0.1 μm to ~0.5 μm.

**Figure 6:**
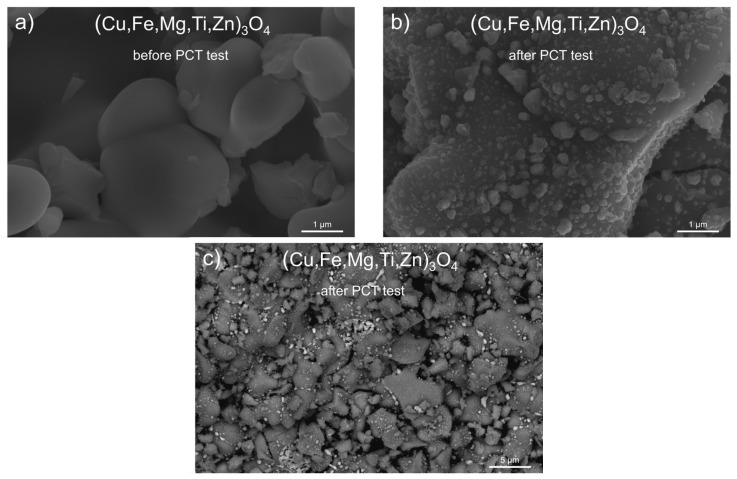
SEM microphotographs for (Cu,Fe,Mg,Ti,Zn)_3_O_4_ samples obtained (**a**) before the PCT test (50,000× magnification), (**b**) after the PCT test (50,000× magnification), and (**c**) after the PCT test (10,000× magnification—CBS detector).

**Figure 7:**
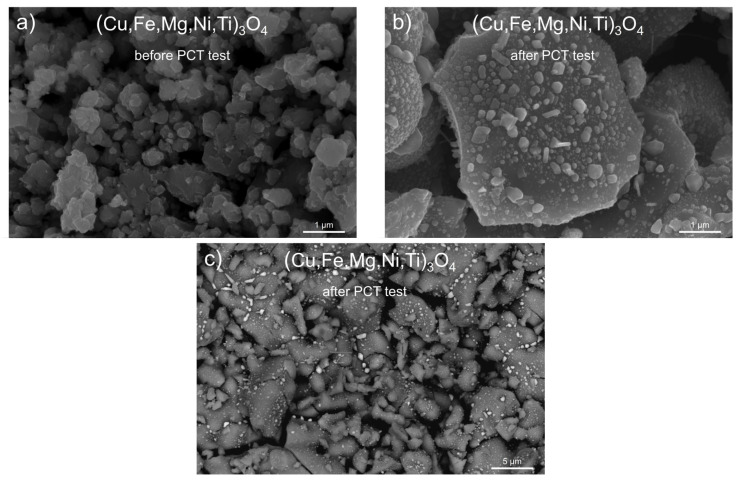
SEM microphotographs for (Cu,Fe,Mg,Ni,Ti)_3_O_4_ samples obtained (**a**) before the PCT test (50,000× magnification), (**b**) after the PCT test (50,000× magnification), and (**c**) after the PCT test (10,000× magnification—CBS detector).

In subsequent Figure 6 and Figure 7, SEM images of copper-containing spinel powders, namely (Cu,Fe,Mg,Ti,Zn)_3_O_4_ and (Cu,Fe,Mg,Ni,Ti)_3_O_4_, are shown, which illustrate their morphology before (Figure 6a and Figure 7a) and after the PCT test carried out at 250 °C (Figure 6b,c and Figure 7b,c).

The grains of both investigated powders before PCT exhibited an irregular shape and usually demonstrated the ability to agglomerate into clusters containing many grains, the sizes of which were between ~0.5 μm and ~5 μm. Fundamental morphological changes in the grains of both (Cu,Fe,Mg,Ti,Zn)_3_O_4_ and (Cu,Fe,Mg,Ni,Ti)_3_O_4_ spinels were observed after the high-pressure hydrogenation process. The grain agglomerate sizes of both studied spinels became smaller, compared to the respective sizes before the PCT test. Signs of cracking were seen on the surfaces of certain grains, which indicated mechanical stresses in the grains as they were penetrated by hydrogen. Another easily observed result of the changes in grain morphology after PCT was the presence of very small precipitates in the form of irregularly shaped particles. The sizes of these particles ranged from ~0.02 μm to ~0.5 μm.

**Figure 8:**
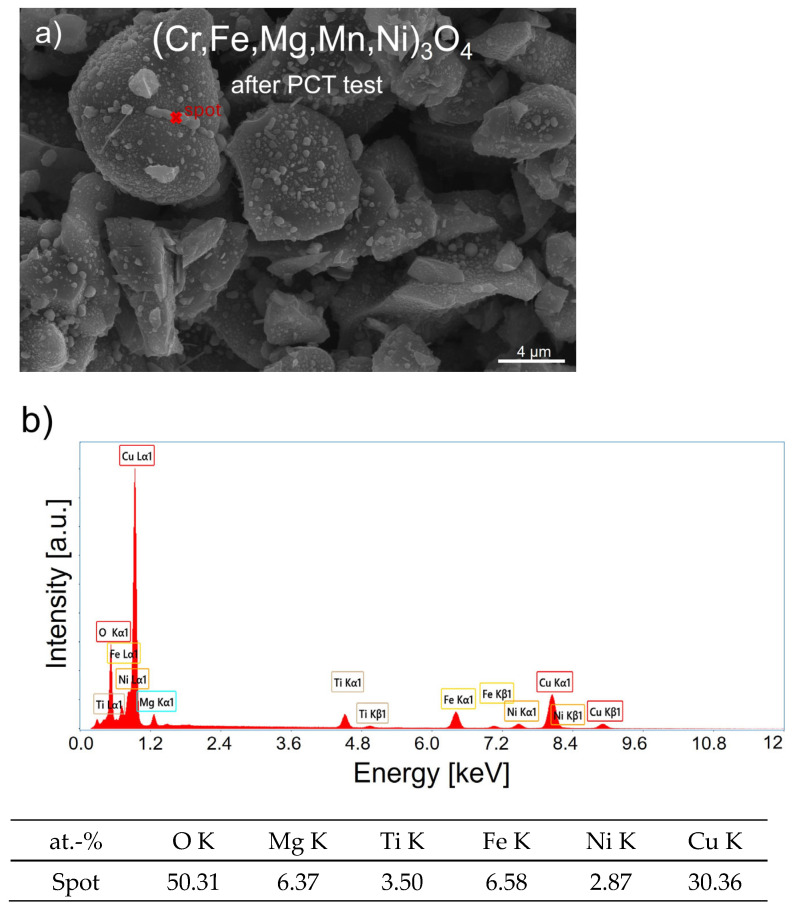
(**a**) SEM microphotograph of (Cu,Fe,Mg,Ni,Ti)_3_O_4_ obtained after the PCT test and (**b**) the EDS quantitative point analysis of area “spot”.

These particles were metallic copper precipitates, which were visible in appropriately magnified SEM microphotographs (Figure 6c and Figure 7c) thanks to the clear contrast between metallic and oxide phases provided by the Circular Backscatter (CBS) detector. These precipitates were identified as copper, based on chemical composition studies performed using EDS point analysis carried out on several of the discussed metallic particles located on both studied (Cu,Fe,Mg,Ti,Zn)_3_O_4_ and (Cu,Fe,Mg,Ni,Ti)_3_O_4_ spinels. In these investigations, the high intensity of the obtained CuLa1 peak confirmed this interpretation. Figure 8 illustrates, as an example, an SEM microphotograph of the (Cu,Fe,Mg,Ni,Ti)_3_O_4_ spinel (Figure 8a), along with a designated region where an EDS spectrum was obtained (Figure 8b). It should be noted that in this sample, as well as the other studied materials, iron concentration was determined as being higher than assumed from the respective stoichiometric compositions. It can be theorized that this was due to the presence of impurities originating from metal mixers and a steel chamber shell.

## 4. Discussion

It was determined from the XRD analysis of powders with the nominal compositions (Cr_0.2_Fe_0.2_Mg_0.2_Mn_0.2_Ni_0.2_)_3_O_4_, (Cu_0.2_Fe_0.2_Mg_0.2_Ti_0.2_Zn_0.2_)_3_O_4_, and (Cu_0.2_Fe_0.2_Mg_0.2_Ni_0.2_Ti_0.2_)_3_O_4_, obtained after high-energy milling (Figure 1), that only applying mechanochemical synthesis to receive single-phase HEOx spinels was inadequate, even after increasing the milling duration to 72 h. Additional high-temperature thermal treatment was necessary to achieve a single-phase or almost single-phase spinel powder. Mechanochemical processes occurring in the system led to the local emission of heat, which is usually used to form metastable phases. In the context of spinel formation, this amount proved to be insufficient to enable single-phase HEOx synthesis at room temperature. The same situation took place in the studies performed by Balcerzak et al. [37], which aimed to use mechanochemical synthesis (MS) to obtain a single-phase (Co,Cu,Mg,Ni,Zn)O HEOx. In that research, additional thermal treatment also became necessary. It was assumed that the high chemical stability of Co_3_O_4_ was responsible for this, as it was the only identified initial component still present in the studied oxide, even after 100 h of the MS process.

By comparing the lattice parameters of the samples before and after hydrogenation (Table 1), a clear increase in the lattice parameter “*a*” of a regular spinel unit cell was observed after hydrogenation, regardless of whether or not additional phases (containing Cu) are present in the studied samples of (Cr,Fe,Mg,Mn,Ni)_3_O_4_, (Cu,Fe,Mg,Ti,Zn)_3_O_4_, and (Cu,Fe,Mg,Ni,Ti)_3_O_4_. This increase was associated with the very likely occurrence of hydrogen incorporated into the material under pressure locating itself in interstitial positions in the spinel structure, which resulted in the relaxation (expansion) of this structure and, subsequently, an increase in interplanar spacing. This, in turn, directly translated to an increase in the lattice parameter value and spinel unit cells. This process similarly took place in all three samples, regardless of whether or not they contained Cu atoms in their structure or if a new additional phase formed after hydrogenation (as previously mentioned). An interesting approach to this issue was the fact that the redistribution of oxygen atoms during the hydrogenation of spinel compounds explained the unusual occurrence of an inverse dependence of the lattice expansion on the hydrogen storage capacity observed for these materials. The hydrogen-induced changes in the crystal structure of Zr_3_MO_x_ (M = Fe, Co, Ni) phases were previously studied [38]. The crystal structure refinement of these hydrides confirmed the oxygen atom redistribution from octahedral to tetrahedral interstices.

Calculated crystallite sizes (Table 1) indicated a decrease in the averaged crystallite size in a given material after high-pressure hydrogenation, compared to the respective samples before the aforementioned process. Taking into account the fact that the lattice parameter “*a*” increased after high-pressure hydrogenation, i.e., the main regular spinel phase unit cells increased in size, it can be assumed that this process caused crystallites of this phase to disintegrate into smaller crystallites. This crystallite size reduction was more pronounced in the (Cu,Fe,Mg,Ti,Zn)_3_O_4_ and (Cu,Fe,Mg,Ni,Ti)_3_O_4_ samples, which contained additional Cu phases after hydrogenation, and CuO and Cu_2_O before hydrogenation in the case of (Cu,Fe,Mg,Ti,Zn)_3_O_4_, as indirectly confirmed by SEM observations performed on the morphologies of these samples (Figure 6 and Figure 7). Introduction of hydrogen atoms into the spinel structure can additionally result in a change in the oxidation state of metal cations; in the case of the studied samples, specifically, copper cations changed from Cu^+2^ or Cu^+1^ to Cu^0^. Along with the reduction of the oxidation state, the Cu ion radii increased, compared to the reduced Cu0, which can consequently lead to a stress increase in the grain structure. To compensate for the resulting stresses, the crystallite grains presumably disintegrated into smaller units. While observing the crystallite dimensions of the studied samples, it was noticeable that the crystallites were much smaller in the cases of the (Cu,Fe,Mg,Ni,Ti)_3_O_4_ and (Cu,Fe,Mg,Ti,Zn)_3_O_4_ samples, in which metallic Cu^0^ appeared as an additional phase.

In the case of the material not containing copper without additional phases before or after hydrogenation, i.e., (Cr,Fe,Mg,Mn,Ni)_3_O_4_, crystallites only decreased slightly, around 20%, compared to their initial dimensions, which was also confirmed by morphology observations (Figure 5). In the case of this sample, the decrease in crystalline dimensions can be associated with the stresses that appeared in the lattice after the lattice parameter of the main phase increased.

The size increase in unit cells of the regular spinel structure in all three samples was accompanied by an increase in lattice strains. These stresses were caused by relative changes (displacement) in atoms in the same ordered domain, e.g., in a crystallite. The displacements can also be described as defects or lattice distortions. As in the case of crystallite size change, the increase in lattice distortions was greater for samples containing additional phases, i.e., (Cu,Fe,Mg,Ti,Zn)_3_O_4_ and (Cu,Fe,Mg,Ni,Ti)_3_O_4_, and smaller in the case of (Cu,Fe,Mg,Ni,Ti)_3_O_4_.

The trends determined for the increases in the regular unit cell lattice parameter of the main phase, along with the simultaneous decrease in crystallite size and increase in lattice distortions after hydrogenation, correlated with one another and agreed with the remaining structural studies carried out on the synthesized materials.

In the case of (Cr,Fe,Mg,Mn,Ni)_3_O_4_, a reaction with hydrogen was not observed, which means that the discussed sample was chemically stable under the influence of hydrogen in high-pressure conditions at 250 °C. Conversely, hydrogen absorption was observed in the two remaining Cu,Fe,Mg,Ti,Zn)_3_O_4_ and (Cu,Fe,Mg,Ni,Ti)_3_O_4_ samples. In both cases, a gradual increase in hydrogen concentration was detected during hydrogenation, where a somewhat higher hydrogen absorption level for (Cu,Fe,Mg,Ti,Zn)_3_O_4_ was also determined. This can be attributed to the additional CuO and Cu_2_O phases in this spinel (Table 1). According to the PCT test performed for pure CuO [36], this oxide demonstrated a high hydrogen absorption level equal to 2.34 wt.% at 100 °C, resulting in its reduction to metallic copper. The same was true for the Cu_2_O compound. This was confirmed by chemical composition results obtained via the EDS analysis carried out on selected areas containing grain agglomerates, on which metallic copper precipitates were visible (Figure 8). Calculated copper mass contents in (Cu,Fe,Mg,Ti,Zn)_3_O_4_ and (Cu,Fe,Mg,Ni,Ti)_3_O_4_ spinels, equal to 17.80 and 17.48 wt.%, respectively, clearly indicated that in addition to copper from the oxides, copper from both spinel structures also underwent reduction. From the data listed in Table 1, it followed that the amounts of reduced copper after high-pressure hydrogenation were 8.9 and 11.9 wt.% for (Cu,Fe,Mg,Ti,Zn)_3_O_4_ and (Cu,Fe,Mg,Ni,Ti)_3_O_4_, respectively. Thus, it can be concluded that the entirety of the copper present in the structures of both investigated spinels did not undergo reduction to its metallic form.

## 5. Conclusions

➢ Single-phase spinel-structured powders were not obtained via the mechanochemical synthesis (MS) of samples with the nominal compositions (Cr_0.2_Fe_0.2_Mg_0.2_Mn_0.2_Ni_0.2_)_3_O_4_, (Cu_0.2_Fe_0.2_Mg_0.2_Ti_0.2_Zn_0.2_)_3_O_4_, and (Cu_0.2_Fe_0.2_Mg_0.2_Ni_0.2_Ti_0.2_)_3_O_4_.
➢ Performing the free sintering procedure on powders after MS at 1000 °C for 3 h in air achieved single-phase (Cr,Fe,Mg,Mn,Ni)_3_O_4_ and (Cu,Fe,Mg,Ni,Ti)_3_O_4_ powders with a spinel structure, and in the case of (Cu,Fe,Mg,Ti,Zn)_3_O_4_, a spinel phase in the amount of 95 wt.% was achieved.
➢ An increase in spinel unit cell lattice parameters was determined for (Cr,Fe,Mg,Mn,Ni)_3_O_4_, (Cu,Fe,Mg,Ti,Zn)_3_O_4_, and (Cu,Fe,Mg,Ni,Ti)_3_O_4_ powders after high-pressure hydrogenation at 250 °C and with a pressure of up to 40 bar.
➢ A decrease in the spinel phase crystallite size and an increase in lattice strains were established in the above-mentioned spinel powders.
➢ Spinels containing copper in their structure, i.e., (Cu,Fe,Mg,Ti,Zn)_3_O_4_ and (Cu,Fe,Mg,Ni,Ti)_3_O_4_, were chemically unstable during high-pressure hydrogenation, due to a partial reduction process accompanied by the release of metallic copper.
➢ Pure hydrogen did not have a notable effect on the structure and morphology of (Cr,Fe,Mg,Mn,Ni)_3_O_4_, which indicates the potential of this ceramic material for future use in hydrogen technologies.

## Figures and Tables

**Table 1:** Structural parameters of the spinel phase in the studied oxides and the secondary phases identified in the studied materials before and after PCT tests at 250 °C.

Sample	(Cr,Fe,Mg,Mn,Ni)_3_O_4_		(Cu,Fe,Mg,Ti,Zn)_3_O_4_		(Cu,Fe,Mg,Ni,Ti)_3_O_4_	
	Before PCT	After PCT	Before PCT	After PCT	Before PCT	After PCT
Parameter “*a*” of the spinel cell [Å]	8.3529	8.3974	8.4348	8.4758	8.3995	8.4366
Identified secondary phases/mass content [wt.%]	-	-	CuO (2.5%) Cu_2_O (1.7%)	Cu (8.9%)	-	Cu (11.9%)
Crystalline size [nm]	65(1)	50(1)	149(1)	60(2)	128(1)	29(1)
Microstresses (×10^−3^)	0.4	2.1	0.2	6.2	0.07	4.2

## Data Availability

The original contributions presented in the study are included in the article, further inquiries can be directed to the corresponding author.
